# Prevalence of Peri-Implant Bone Loss in Patients with a History of Periodontal Disease: A Retrospective Study

**DOI:** 10.4317/jced.63435

**Published:** 2025-11-30

**Authors:** Greta Ognyanova-Toleva, Rocío Cascos-Sánchez, José Luis Antonaya-Martín, Pablo Lastra-Prados, Noelia Rivas-Martín, Diego Gómez-Costa

**Affiliations:** 1 Master’s Degree in Implant-Supported Prostheses, Rey Juan Carlos University (URJC); 2 Lecturer in the Master’s Programme in Implant-Supported Prostheses, URJC; 3 Doctor of Dentistry, Director of the Master’s Programme in Implant-Supported Prostheses, URJC

## Abstract

**Background:**

The primary aim of this retrospective study was to determine the prevalence of peri-implant bone loss in patients with a documented history of periodontal disease. The secondary aim was to evaluate the influence of various demographic, systemic, and prosthetic variables on marginal bone level changes.

**Material and Methods:**

A retrospective analysis was conducted on a cohort of 261 patients treated with implant-supported prostheses at the Rey Juan Carlos University Clinic between 2018 and 2023. Anonymised data were extracted from clinical records and radiographic archives. Variables analysed included age, sex, smoking status, diabetes mellitus, type of prosthesis, use of transepithelial abutments, implant brand, and periodontal history. Bone loss was categorised as none, &amp;#x0003C;1.5 mm, or &amp;#x0003E;1.5 mm. Statistical analysis employed descriptive statistics, Kolmogorov-Smirnov tests for normality, and non-parametric tests (Kruskal-Wallis and Chi-square) using IBM SPSS Statistics, version 25.0.

**Results:**

The overall prevalence of peri-implant bone loss was 24.5%. Within the cohort with a periodontal history (60.5% of patients), the prevalence was 14.5%. Statistical analysis revealed that only the use of a transepithelial abutment (p=0.000) and the implant brand (p=0.001) demonstrated a statistically significant influence on bone loss levels. No significant associations were found with a history of periodontitis alone, age, sex, diabetic status, smoking habit, or type of prosthetic restoration.

**Conclusions:**

Within the limitations of this study, a history of periodontal disease was not a determining factor for increased peri-implant bone loss. The findings underscore the critical importance of prosthetic design, specifically the use of transepithelial abutments, and implant selection in ensuring favourable medium-term outcomes. These results suggest that stringent periodontal therapy and maintenance protocols may mitigate the inherent risks in this patient population.

## Introduction

The utilisation of dental implants represents a cornerstone of contemporary restorative dentistry, offering a predictable and highly successful solution for the rehabilitation of partially and fully edentulous patients. Reported long-term survival rates routinely exceed 95%, affirming their status as a first-line treatment modality (1 , 2). However, the burgeoning application of implant therapy has been paralleled by an increased incidence of biological complications, chiefly peri-implant mucositis and its more destructive counterpart, peri-implantitis (3). Peri-implantitis is an inflammatory lesion of microbial aetiology that affects the soft and hard tissues surrounding an osseointegrated Peri-implantitis is an inflammatory lesion of microbial aetiology that affects the soft and hard tissues surrounding an osseointegrated implant, leading to progressive marginal bone loss and, if left untreated, eventual implant failure (4 , 5). A key metric in the diagnosis and monitoring of peri-implant health is marginal bone level change. Whilst a degree of initial bone remodelling following prosthetic loading is considered physiological, often cited as up to 1.5-2.0 mm in the first year, ongoing bone loss is a hallmark of pathology (6 , 7 , 8). Patients with a history of periodontitis have been consistently identified in the literature as a high-risk group for the development of peri-implant diseases (9 , 10). The proposed mechanisms for this increased susceptibility include the persistence of a dysbiotic oral microbiome, a heightened individual inflammatory response, and genetic predispositions (11). Systematic reviews and meta-analyses have suggested that these patients exhibit lower implant survival rates and greater marginal bone loss compared to periodontally healthy individuals (12 , 13). Nevertheless, the evidence is not entirely unequivocal. Outcomes appear to be influenced by a complex interplay of factors beyond periodontal history, including the quality of periodontal therapy prior to implant placement, the rigour of a lifelong supportive maintenance programme, surgical technique, implant macro- and micro-design, and prosthetic components (14 , 15). The debate thus persists regarding the absolute weight of periodontal history as an independent risk factor, particularly in cohorts receiving structured care within a university or specialist setting. This study aims to contribute robust clinical data to this debate through a retrospective analysis of a well-characterised patient cohort. The null hypothesis is that there is no statistically significant difference in the prevalence of peri-implant bone loss between patients with and without a history of periodontal disease.

## Material and Methods

**Figure 1:**
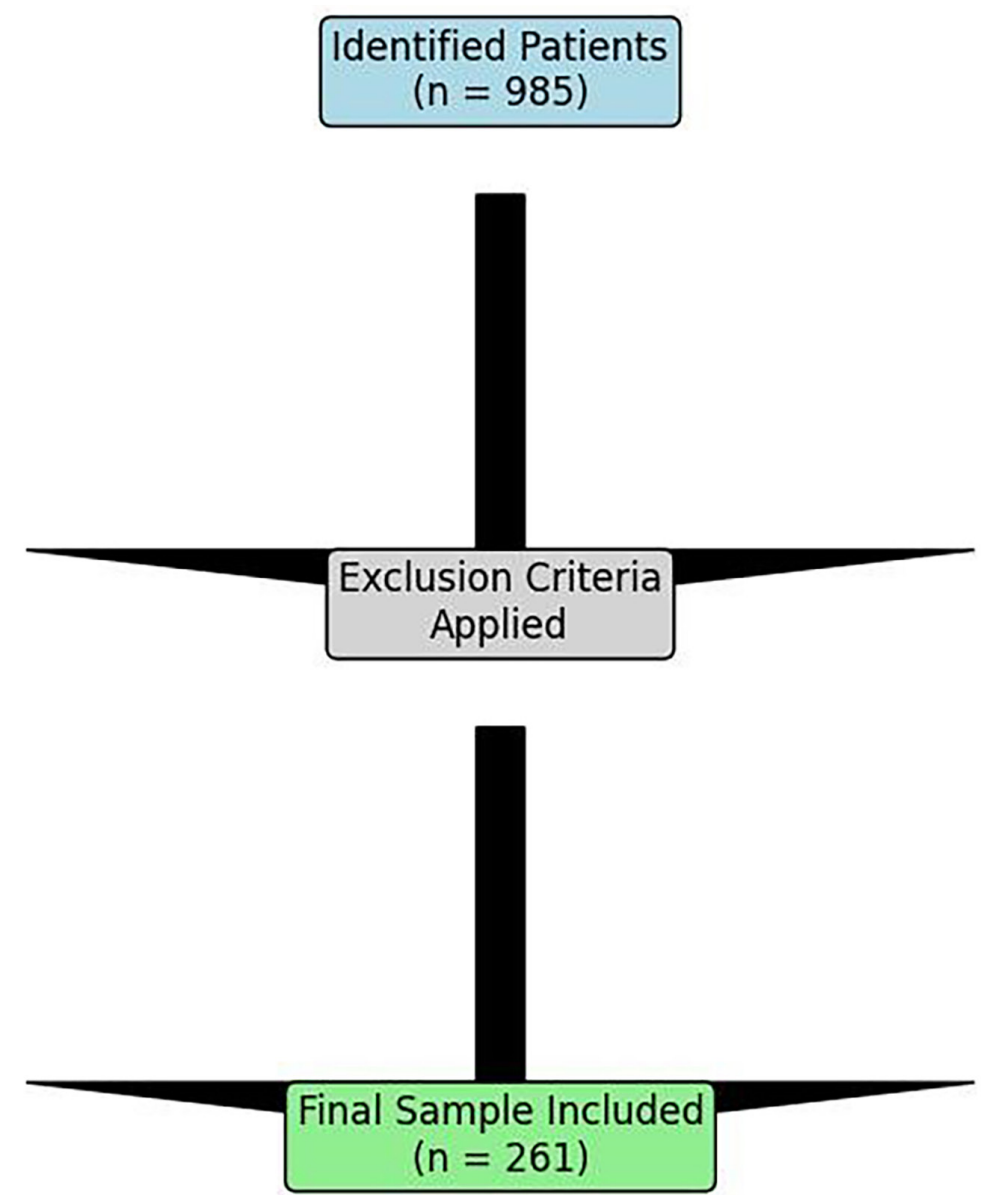
Sample Selection Flowchart.

1. Study Design and Ethical Approval This investigation was designed as a single-centre, retrospective, observational cohort study. The study protocol received approval from the relevant institutional review board of the Rey Juan Carlos University. All procedures were conducted in accordance with the ethical standards of the 1964 Helsinki declaration and its later amendments. Patient data were anonymised at source prior to analysis, with access restricted to the principal investigators. 2. Patient Selection and Eligibility Criteria The initial screening identified 985 patients who had undergone implant therapy at the Rey Juan Carlos University Clinic between January 2018 and December 2023, (Fig. 1).

1

**Figure 2:**
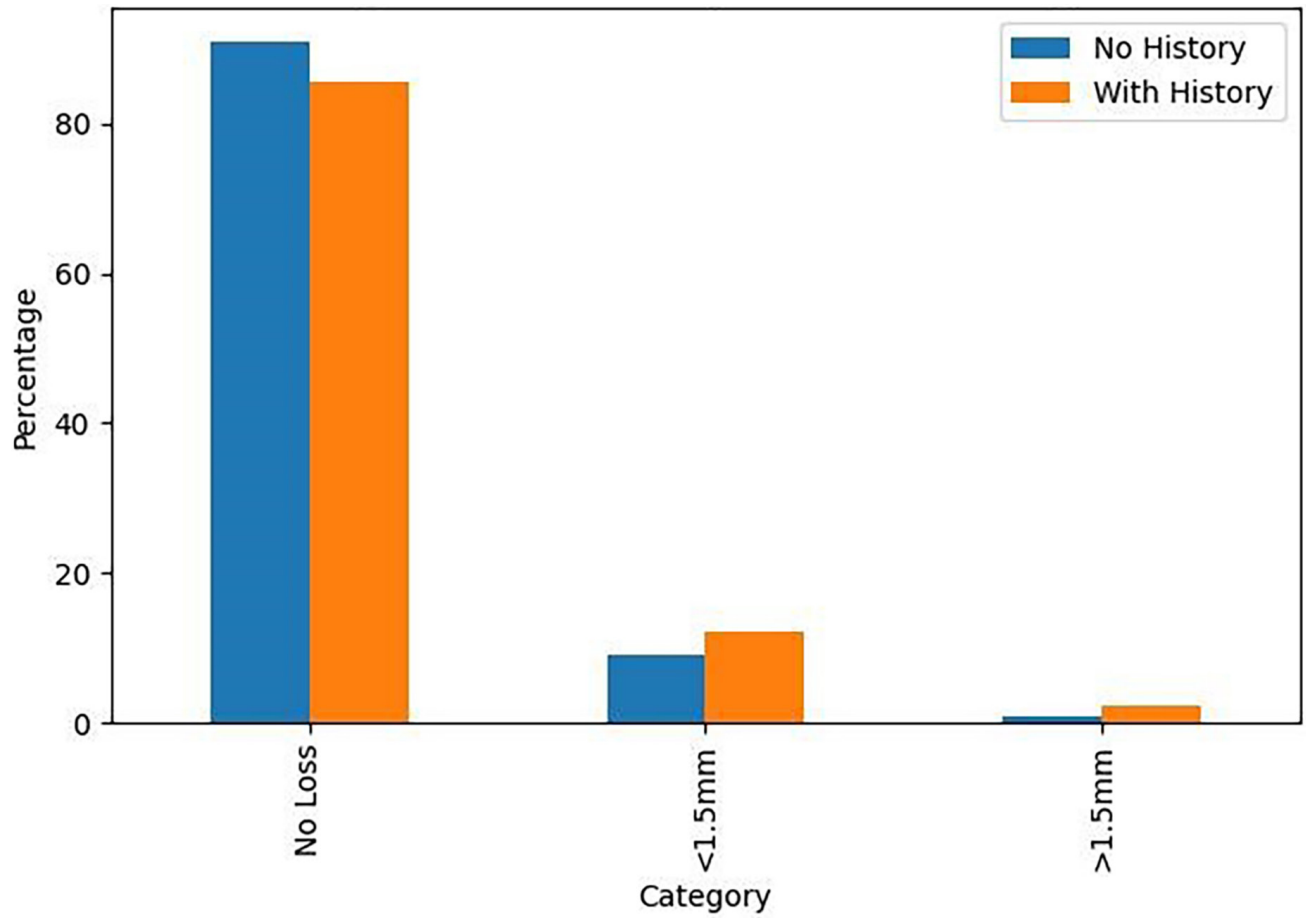
Bone Loss by Periodontal History.

**Figure 3:**
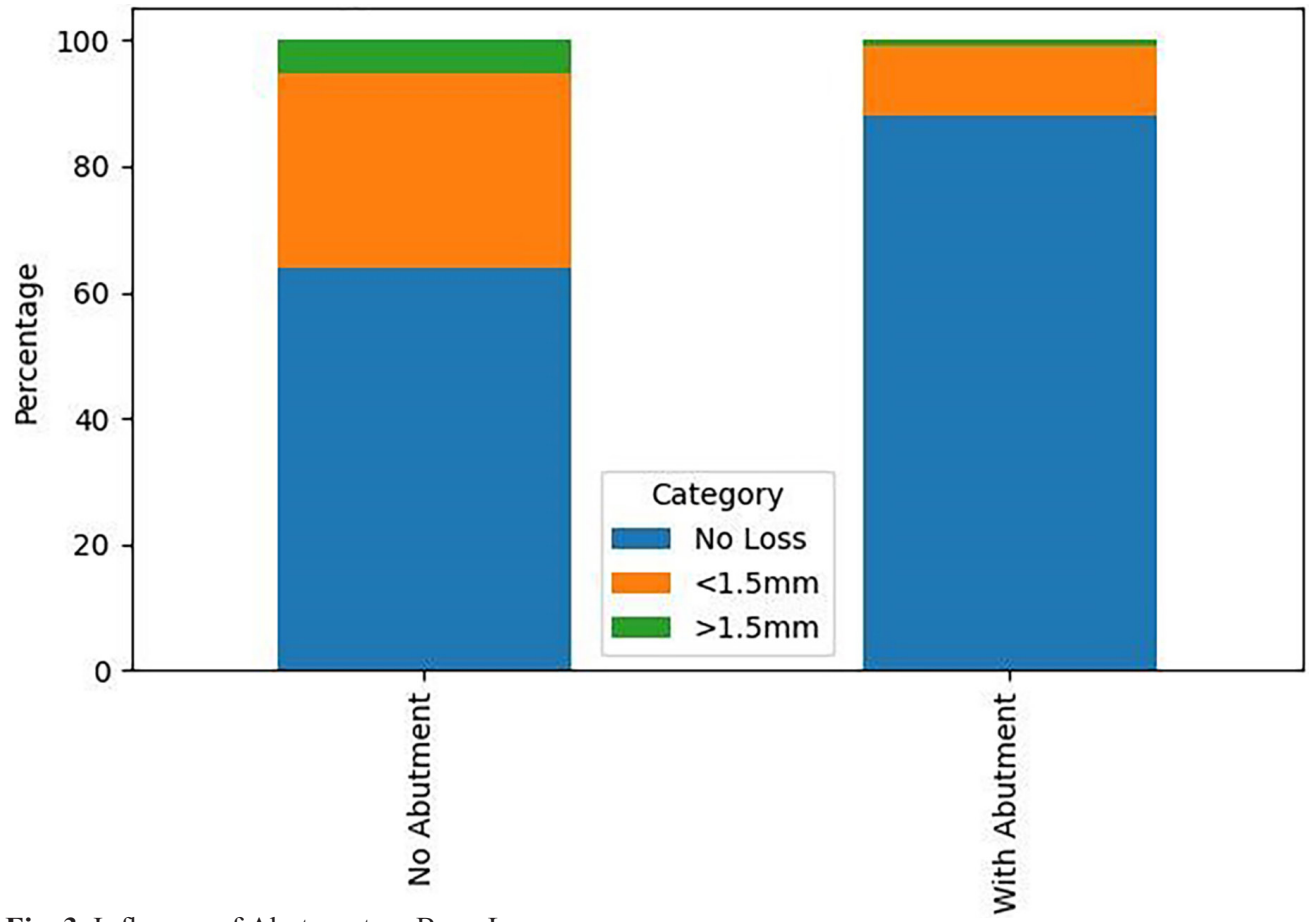
Influence of Abutment on Bone Loss.

The application of inclusion and exclusion criteria yielded a final sample size of 261 patients. Inclusion Criteria comprised: (1) Patients with implant-supported prostheses delivered at the university clinic; (2) Availability of complete clinical records and radiographic series (including either panoramic radiographs or parallel periapical radiographs) taken at the time of prosthetic delivery and at a minimum of one-year follow-up; (3) For patients with a history of periodontitis, documented completion of active periodontal therapy prior to or concurrent with implant treatment; (4) Attendance at a minimum of one annual maintenance visit. Exclusion Criteria were: (1) Implants placed outside the university clinic; (2) Records predating 2018 due to incomplete digitalisation; (3) Incomplete or missing clinical records; (4) Lack of comparable radiographic follow-up. 3. Data Collection and Variables Data were meticulously extracted from the clinic's digital management system (Gestiona Klinikare 2022 (c)) and radiographic software (CareStream Dental). Each patient was assigned a unique numerical code to ensure anonymity. The following variables were recorded in a standardised Microsoft Excel® spreadsheet: Dependent Variable: Peri-implant bone loss (categorised as: No bone loss; Bone loss &lt; 1.5 mm; Bone loss &gt; 1.5 mm). Measurement was performed mesially and distally to each implant on serial radiographs, (Figs. 2,3).

2

3

**Figure 4:**
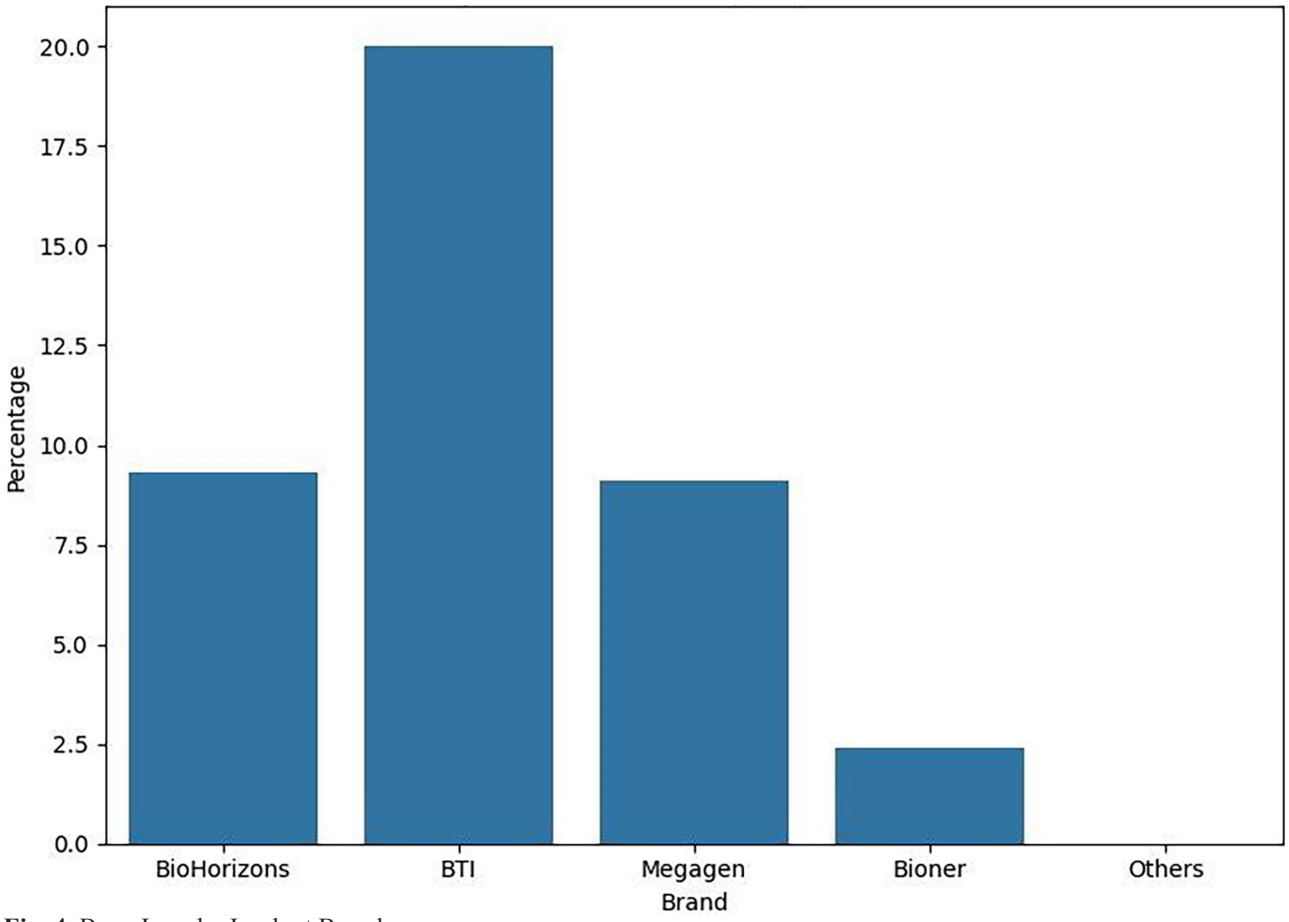
Bone Loss by Implant Brand.

Independent Variables: Age (years); Sex (Male/Female); Nationality; Smoking habit (Non-smoker/Smoker); Diabetic status (Non-diabetic/Diabetic); Periodontal history (Yes/No); Type of implant-supported prosthesis (Single crown; Fixed bridge; Hybrid prosthesis; Bar-retained overdenture); Use of a transepithelial abutment (Yes/No); Implant brand. 4. Statistical Analysis Statistical analysis was performed using IBM SPSS Statistics for Windows, Version 25.0 (Armonk, NY: IBM Corp). Descriptive statistics were computed for all variables. Given the sample size (n=261 &gt; 50), the assumption of normality was assessed using the Kolmogorov-Smirnov test. As the data for the quantitative variable (age) and the ordinal bone loss variable did not follow a normal distribution (p&lt;0.05), non-parametric tests were employed. The Kruskal-Wallis H test was used to analyse the relationship between bone loss and periodontal history, and between bone loss and age. The Chi-square test was applied to assess the associations between bone loss and the qualitative variables (sex, smoking, diabetes, prosthesis type, abutment use, implant brand). A confidence level of 95% was set for all analyses, with a p-value 0.05 deemed statistically significant, (Fig. 4).

4

## Results

1. Descriptive Statistics The final study cohort consisted of 261 patients. The sample was slightly predominated by females (143 patients, 54.8%) over males (118 patients, 45.2%). The mean age was 60.33 years (SD ±10.76), with a range from 34 to 82 years. A majority of patients (158, 60.5%) had a documented history of periodontal disease, while 103 (39.5%) had no such history. Regarding prosthetic rehabilitation, single crowns were the most common restoration (146, 55.9%), followed by fixed partial dentures (107, 41.0%). Hybrid prostheses and bar-retained overdentures were less frequent (5 and 3 patients, respectively). Transepithelial abutments were used in 126 patients (48.3%). The majority of patients were non-smokers (209, 80.1%) and non-diabetic (241, 92.3%). The most frequently used implant brand was Alphabio (23.7%). Concerning the primary outcome, 197 patients (75.5%) exhibited no discernible bone loss, 56 (21.4%) had bone loss less than 1.5 mm, and 8 (3.1%) had bone loss exceeding 1.5 mm. 2. Inferential Statistics The central finding of this study was that a history of periodontal disease was not a statistically significant predictor of peri-implant bone loss (Kruskal-Wallis, p=0.898). The prevalence of bone loss in the periodontally compromised group was 14.5% (12.2% &lt;1.5mm; 2.3% &gt;1.5mm), compared to 10.0% (9.2% &lt;1.5mm; 0.8% &gt;1.5mm) in the periodontally healthy group. Conversely, two factors demonstrated a highly significant influence: Use of a Transepithelial Abutment: This variable showed a profoundly significant association with bone loss (Chi-square, p=0.000). A markedly higher proportion of patients rehabilitated without an abutment experienced bone loss (18.8% vs. 5.8% in the abutment group for loss &lt;1.5mm; 2.7% vs. 0.4% for loss &gt;1.5mm). Implant Brand: The choice of implant system also had a significant impact on bone loss levels (Chi-square, p=0.001). Brands such as BioHorizons and Biomet 3i were associated with a higher incidence of bone loss compared to others. No statistically significant associations were found between peri-implant bone loss and the variables of age (p=0.980), sex (p=0.671), smoking status (p=0.427), diabetic status (p=0.148), or type of prosthetic restoration (p=0.903).

## Discussion

This retrospective analysis of 261 patients rehabilitated with dental implants provides compelling evidence that challenges the deterministic view of periodontal history as the primary driver of peri-implant bone loss. Our results indicate that whilst a history of periodontitis is a consideration, its influence can be effectively mitigated by meticulous attention to prosthetic design and implant selection within a structured maintenance programme. The null hypothesis that no significant difference exists in bone loss between patients with and without a periodontal history is accepted. This finding appears to contradict several previous meta-analyses and systematic reviews (12 , 13). However, a crucial differentiating factor of our cohort is that all patients, particularly those with a periodontal history, were treated and maintained under a strict university clinic protocol. This protocol mandates definitive periodontal treatment prior to implant placement and enforces a rigorous, lifelong schedule of supportive periodontal and peri-implant therapy. This suggests that the elevated risk associated with periodontitis is not immutable but can be modified by the quality of care. Our findings align with emerging literature that emphasises the modifiability of risk through comprehensive management (14 , 15 , 19). The profound significance of the transepithelial abutment is a pivotal finding. The use of such abutments is thought to improve biomechanical stress distribution, facilitate superior soft tissue attachment and sealing, and allow for a more passive and hygienic emergence profile of the final restoration (16 , 20). This result has direct and immediate implications for clinical treatment planning, strongly advocating for the routine use of custom or stock transepithelial abutments in implant rehabilitations. Similarly, the significant variation in outcomes based on implant brand underscores the importance of evidence-based selection of implant systems. Differences in implant neck design, surface topography, and connection type can profoundly influence crestal bone maintenance (16 , 21). This finding advises clinicians to be discerning in their choice of implant system, prioritising those with robust long-term clinical data. The lack of significance for other variables, such as smoking and diabetes, may be attributable to the relatively low prevalence of these conditions in our sample or, again, to the controlling effect of the strict maintenance protocol (22). Limitations The limitations inherent to the retrospective design must be acknowledged. The reliance on existing clinical records introduces potential for heterogeneity in data recording. Radiographic assessment, whilst standardised, was based on 2D imaging, which precludes assessment of buccal and lingual bone levels. Furthermore, the definition of periodontal history, though based on clinic records, could lack the granularity of a prospective periodontal diagnosis. Finally, the sample size for certain variables (e.g., hybrid prostheses, specific implant brands) was small, limiting the power of sub-group analyses.

## Conclusions

Within the constraints of this study, the following conclusions can be drawn: The prevalence of peri-implant bone loss in patients with a history of periodontal disease was 14.5% after a minimum of one year in function. A history of periodontal disease was not found to be a statistically significant factor for increased peri-implant bone loss in this patient cohort receiving structured care. The use of a transepithelial abutment and the selection of implant brand were identified as the only factors with a statistically significant influence on marginal bone levels. These results highlight the critical importance of meticulous prosthetic design, evidence-based implant selection, and, most importantly, the execution of definitive periodontal therapy and unwavering adherence to a supervised maintenance programme for the long-term success of implant therapy in periodontally compromised patients (19 , 23).

## Data Availability

The datasets used and/or analyzed during the current study are available from the corresponding author.
